# Design and Validation of an Active Headrest System with Integrated Sensing in Rear-End Crash Scenarios

**DOI:** 10.3390/s25144291

**Published:** 2025-07-09

**Authors:** Alexandru Ionut Radu, Bogdan Adrian Tolea, Horia Beles, Florin Bogdan Scurt, Adrian Nicolaie Tusinean

**Affiliations:** 1 Department of Automotive and Transport, Faculty of Mechanical Engineering, Transilvania University of Brasov, 500036 Brasov, Romania; alexandru.radu@unitbv.ro; 2 Department of Mechanical Engineering and Automotive, Faculty of Managerial and Technological Engineering, University of Oradea, 410087 Oradea, Romania; horia.beles@uoradea.ro (H.B.); scurt.florinbogdan@didactic.uoradea.ro (F.B.S.); 3 Advanced Mechatronik Technologies SRL, 500025 Brasov, Romania; adriantusinean@gmail.com

**Keywords:** mathematical model, active headrest, integrated sensor, simulation, multibody, Simscape multibody, dynamic simulation, crash test

## Abstract

Rear-end collisions represent a major concern in automotive safety, particularly due to the risk of whiplash injuries among vehicle occupants. The accurate simulation of occupant kinematics during such impacts is critical for the development of advanced safety systems. This paper presents an enhanced multibody simulation model specifically designed for rear-end crash scenarios, incorporating integrated active headrest mechanisms and sensor-based activation logic. The model combines detailed representations of vehicle structures, suspension systems, restraint systems, and occupant biomechanics, allowing for the precise prediction of crash dynamics and occupant responses. The system was developed using Simscape Multibody, with CAD-derived components interconnected through physical joints and validated using controlled experimental crash tests. Special attention was given to modelling contact forces, suspension behaviour, and actuator response times for the active headrest system. The model achieved a root mean square error (RMSE) of 4.19 m/s^2^ and a mean absolute percentage error (MAPE) of 0.71% when comparing head acceleration in frontal collision tests, confirming its high accuracy. Validation results demonstrate that the model accurately reproduces occupant kinematics and head acceleration profiles, confirming its reliability and effectiveness as a predictive tool. This research highlights the critical role of integrated sensor-actuator systems in improving occupant safety and provides a flexible platform for future studies on intelligent vehicle safety technologies.

## 1. Introduction

Motor vehicle collisions, particularly frontal and rear-end impacts, continue to pose a significant risk to vehicle occupants and represent a major concern for automotive safety research.

According to global accident statistics, rear-end and frontal impacts remain among the most frequent types of vehicular collisions, often leading to whiplash and head–torso-related injuries. In the European context, rear-end collisions account for over 30% of road traffic incidents with injuries, while frontal collisions are associated with high fatality rates due to high-speed impacts [1]. These types of accidents are strongly correlated with occupant injury mechanisms, which this study aims to address by using biomechanical simulations. A detailed statistical review and classification of such accidents is available in [2].

Understanding the kinematics and dynamics of occupants during such collisions is critical for enhancing vehicle design and improving occupant protection systems.

Mathematical modelling has proven to be a powerful tool in this field, offering detailed insights into the behaviour of vehicle structures and human biomechanics during crash events [3,4,5].

Among these approaches, multibody system (MBS) models have gained prominence due to their ability to accurately simulate complex interactions between the vehicle, restraint systems, and human body segments [6,7,8]. Recent advancements have highlighted the effectiveness of MBS models validated by experimental crash tests, providing robust frameworks for analyzing occupant kinematics and injury mechanisms. For instance, Ionut et al. (2017) developed a frontal collision model validated with crash tests, while Radu et al. (2021) extended this methodology to side impacts [3,4].

Furthermore, Xu et al. (2023) introduced a refined three-dimensional multibody dynamics model for single-vehicle crashes against rigid barriers [5]. Complementary studies by Ryu et al. (2023) and An et al. (2023) expanded the application of MBS to multibody contact dynamics and vehicle–infrastructure interactions, respectively [6,7,8]. A growing body of research has also explored active safety systems aimed at mitigating occupant injuries. Kim et al. (2020) analyzed the behaviour of active and proactive headrests during low-speed rear-end collisions, demonstrating the benefits of dynamic head support [9]. Zhang et al. (2023) and Vychytil et al. (2020) developed and validated active headrest mechanisms to improve occupant biomechanics in rear-end impacts [10,11]. Other studies, such as He et al. (2022, 2024) and Yan and Chen (2023), proposed semi-active seat designs and adaptive headrest systems that respond to occupant posture and crash dynamics [12,13,14]. Furthermore, Lin et al. (2023) applied finite element modelling to study head–neck biomechanical responses during impacts, highlighting alternative high-fidelity modelling techniques [14]. Liu and Zhang (2024) enhanced the capabilities of multibody dynamic simulations specifically for rear-end collisions [15], while Yuan and Li (2023) focused on sensor-based active headrest deployment to improve real-time injury mitigation [16]. Additionally, complementary research has emphasized the role of road surface effects [17], machine learning-based crash severity estimation [18], and the influence of seat configurations on injury risk [19], underlining the multidisciplinary nature of modern vehicle safety studies [20].

Further advancements in occupant protection systems have focused on innovations in airbag and seatbelt technologies. Kitagawa et al. (2012) developed an enhanced airbag system specifically designed for far-side impact protection, addressing occupant movement in lateral collisions [21].

Similarly, Mizuno (2015) evaluated advanced seatbelt systems that demonstrated significant reductions in chest injury risks during frontal crashes, underlining the critical role of restraint system design [22,23,24]. Research on contact dynamics modelling has also gained importance in accident analysis.

Benea and Soica (2023) emphasized the significance of accurately simulating the contact phase in vehicle–pedestrian collisions, providing valuable insights for improving safety measures and reconstruction methodologies [25]. Additionally, Soica (2024) investigated the forces transmitted through pretensioner tubes during collisions, demonstrating how mechanical modelling can aid in accident reconstruction and injury analysis [26]. These studies collectively highlight the essential role of precise mathematical modelling, occupant–restraint interaction analysis, and advanced safety system development in improving overall vehicle safety performance.

Recent advances in Micro-Electro-Mechanical Systems (MEMS) technology have enabled the development of highly sensitive, compact, and cost-effective acceleration sensors suitable for automotive safety applications. MEMS accelerometers are widely used in vehicle crash-detection systems due to their ability to rapidly measure dynamic changes in acceleration with high precision [27,28]. In the context of active headrest systems, acceleration sensors play a critical role by detecting the onset of rear-end collisions. Upon registering a sudden longitudinal acceleration that exceeds a predetermined threshold—typically around 50 m/s^2^—the MEMS sensor triggers the activation mechanism, propelling the headrest forward to mitigate the relative motion between the head and torso [17,29]. The compact design and robustness of MEMS sensors allow for seamless integration into vehicle structures, such as within the seat back or vehicle frame. Their high sampling rates and resilience to vibration and temperature variations ensure reliable operation, even under severe crash conditions [27,28]. The integration of sensor-based logic into the active headrest system thus provides a significant enhancement to occupant protection, reducing the risk of whiplash injuries by supporting the occupant’s head earlier in the collision event [17,29].

Building upon this foundation, this work proposes an enhanced mathematical multibody model specifically designed for analyzing rear-end collisions involving integrated active headrest systems and embedded sensor-based logic. By incorporating realistic contact algorithms and detailed occupant kinematics, the model aims to predict crash outcomes and occupant responses with greater precision, addressing the limitations of current models, which often lack detailed contact mechanics and fail to accurately capture the complex kinematics of the head and neck during rear-end collisions.

The key contributions of this study include the following:

- The development of a comprehensive multibody simulation model integrating active headrest mechanisms.
- The validation of the model against experimental crash data.
- The demonstration of its potential for optimizing occupant protection systems and supporting the design of advanced automotive safety features.

This model offers a robust, flexible tool for simulating rear-end crash dynamics and evaluating the performance of sensor-driven active safety technologies, bridging the gap between traditional MBS techniques and emerging intelligent vehicle systems.

## 2. Background

An earlier version of the model was developed in analytical form, with the equations of motion derived and solved using the ODE45 numerical solver in MATLAB 2024b [30]. This initial model was designed to investigate frontal collision dynamics and occupant kinematics. However, due to its simplified structure, it exhibited limitations in versatility and the ability to represent more complex crash scenarios [3]. Despite these constraints, the results demonstrate that key dynamic parameters could be estimated with good accuracy using a relatively simple analytical approach.

**Figure 1:**
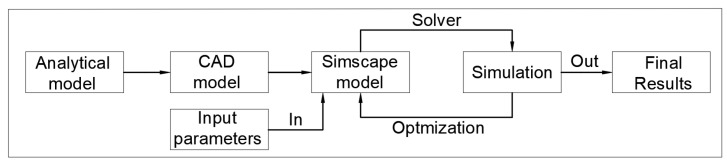
System diagram for model development.

Following the positive results obtained using the initial analytical model, the next development phase focused on creating a complete multibody system capable of producing highly accurate results when compared with experimental crash test data. To illustrate the workflow for building and optimizing the multibody system, a schematic diagram has been prepared, as shown in Figure 1. This diagram outlines the sequential process, starting from the analytical model, progressing through CAD modelling, and culminating in a validated Simscape multibody simulation environment, with iterative optimization based on input parameters and simulation outputs.

The second development step involved creating a detailed CAD model of the components, using the real-world dimensions of both the vehicles and the occupant body segments. The resulting CAD model was then imported into Simscape Multibody, where additional information was incorporated, including mechanical joints, contact force models, body positioning, solver configurations, and dynamic input parameters. The multibody model was constructed using the physical network approach in Simscape, which employs physical blocks and realistic connections to ensure the accurate representation of the system’s behaviour and practical usability. The numerical solver selected by the software was ODE3 (Bogacki–Shampine method), automatically determined based on the model’s configuration, the specified initial conditions, and the defined simulation time step.

## 3. Methodology

### 3.1. Model Description

**Figure 2:**
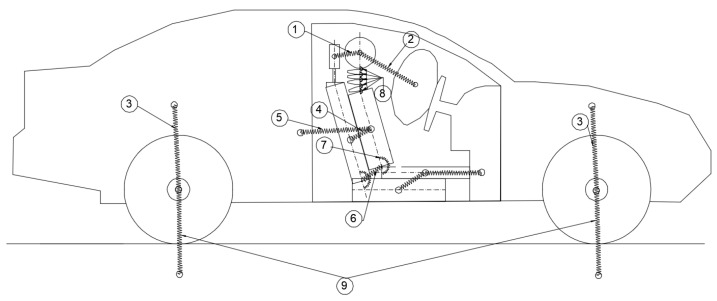
Model connections and relationships between bodies.

The multibody system model was developed using CAD-generated parts, with each component connected using appropriate mechanical joints to realistically replicate the physical interactions between the occupant and the vehicle structure. The initial step in the modelling process involved creating a two-dimensional schematic diagram to illustrate the connections between the bodies and the types of stiffness elements (springs and dampers) implemented in the system. Figure 2 presents this schematic representation, highlighting the interaction points between the various components and specifying whether the connection is modelled as a translational spring–damper (rigid connection) or a rotational spring–damper (torsional connection). This schematic formed the foundation for constructing the complete multibody model in the Simscape environment.

The experimental crash tests used for model validation were conducted at the High-Tech Product Development Research and Innovation Institute of Transilvania University of Brașov. To capture acceleration data, two types of acquisition systems were employed. A PIC DAQ system was mounted in the front floor section of the vehicle to measure accelerations at the vehicle’s centre of mass. Additionally, for occupant motion analysis, a PicDAQ MT5 system equipped with tri-axial accelerometers was installed in the dummy’s torso and head. These sensors recorded accelerations along the X, Y, and Z axes with a sampling rate of up to 2 kHz, and data were logged to an SD card for further processing using PocketDAQ 1.0 software.

To support the kinematic analysis of the occupant, high-speed video recording equipment was deployed, including Casio Exilim EX-F1 and Fastec Hispec 5 cameras, capable of capturing up to 1400 frames per second. Still image documentation was performed using Nikon AW 100 and Samsung Galaxy S5 cameras. In addition, a DS5 GPS system was used to record vehicle speed, position, and impact velocity, utilising a GPS receiver connected via RS 232-USB interface to a notebook.

The testing area was secured using safety protocols such as isolation of the test zone, reliable towing and braking systems, and remote braking controls. The detailed setup ensured reproducibility and safe execution of crash scenarios that served to validate the developed multibody simulation model.

The complete model consists of several interconnected components designed to accurately replicate the dynamics of a rear-end collision scenario. The vehicle structure is modelled as a single rigid body, connected to the four wheels through suspension systems represented by prismatic joints with spring–damper elements (presented in Table 1).

The seat assembly is divided into two distinct components: the lower seat and the backrest. These two elements are connected by a revolute joint equipped with a torsional spring–damper, allowing for the realistic flexion of the seatback during impact.

The occupant is modelled as a simplified anthropomorphic structure without limbs, consisting of the lower body, torso, neck, and head. The neck itself is articulated using four sequential vertebrae, each connected by revolute joints to capture the flexibility of the cervical spine. All segments of the occupant are connected through revolute joints, enabling realistic relative rotations between body parts.

The occupant is positioned on the seat via direct body contacts, simulating gravitational preload and support. Additionally, the torso and lower body are connected to the vehicle body via linear springs, representing seatbelt forces that restrain the occupant during impact. Contact interactions between the lower body, torso, and head with the dashboard are also modelled. The airbag is represented as a single non-deformable body; although no visual deformation is simulated, dynamic interaction is modelled via contact forces. The deceleration effect of the airbag on the head is computed based on predefined stiffness and damping contact parameters. This detailed setup ensures that the occupant dynamics, restraint effects, and head–dashboard–airbag interactions are realistically captured within the simulation framework.

**Figure 3:**
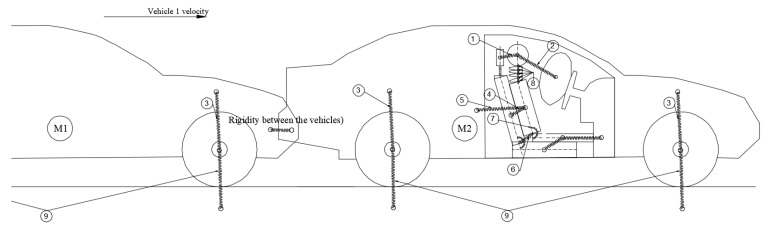
Collision model between the two vehicles.

To expand the model and simulate rear-end collision dynamics more realistically, a second vehicle was incorporated into the system. A contact connection was defined between the front structure of the first vehicle and the rear structure of the second vehicle, enabling the evaluation of impact forces and deformation behaviour at the point of collision. This addition allows for the accurate modelling of the force transmission between the two vehicles during a rear-end impact and ensures that the dynamic responses of both vehicles are captured within the simulation framework. In this study, a simplified modelling approach is adopted where both vehicles are assumed to have identical geometry and dimensions. This assumption facilitates the analysis of occupant dynamics while focusing on the interaction forces during rear-end impacts (see Figure 3).

**Figure 4:**
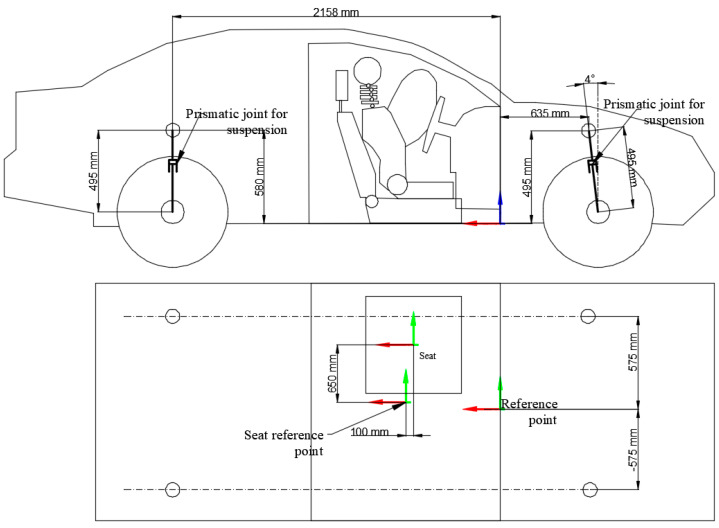
Model dimensions with respect to the reference points.

Figure 4 presents the principal dimensions of the vehicle model along with the reference points used for positioning and alignment during simulation. The side view shows the longitudinal and vertical distances between key structural points, including the location of the prismatic joints used to simulate suspension movement. The suspension joints are positioned to accurately replicate the vehicle’s real suspension geometry, and their relative heights and distances from the vehicle centre are specified.

Figure 4 highlights the vehicle’s main reference point, which serves as a fixed origin for global positioning within the simulation environment. In the top view, the layout of the seat relative to the vehicle structure is illustrated, including the seat reference point positioned 100 mm rearward of the global reference point, ensuring the accurate placement of the occupant model within the cabin. The distances between the suspension mounting points across the vehicle width are also detailed, supporting the symmetrical construction of the chassis in the model.

This dimensional accuracy was essential for ensuring that the multibody system behaves realistically during impact scenarios, particularly in replicating suspension kinematics and occupant–seat interactions. The principal dimensions of the vehicle model and the associated reference points are presented in Figure 4. The side view details the longitudinal and vertical distances between key structural points, including the placement of the suspension prismatic joints and the inclination of the front suspension system. The top view illustrates the seat positioning relative to the main reference point of the vehicle, with the seat reference point positioned 100 mm rearward of the global reference. Symmetrical distances between suspension mounting points are also shown, ensuring consistent chassis construction. Accurate definition of these dimensions and reference points was essential to ensure the realistic simulation of suspension kinematics, occupant seating position, and impact dynamics.

### 3.2. Contact Modelling

Each body in motion within the model is governed by its own differential equation of motion. The principle underpinning the model is based on the classical sprung mass system, described using the following general form [31]:

(1) $$M\frac{d^{2}x}{dt^{2}}+C\frac{dx}{dt}+Kx=F$$

where

- *x* is the vector of displacements and rotational angles of the bodies;
- *M* is the mass matrix;
- *C* is the damping matrix;
- *K* is the stiffness matrix;
- *F* is the vector of external forces acting on the system.

This general equation is applied to all moving components within the model and the contact interactions between vehicles and between occupants and interior structures. It provides a unified framework for modelling both translational and rotational dynamics, as well as contact and spring–damper behaviours throughout the system.

### 3.3. Model Equations

Following the general formulation of the dynamic system, the specific equations of motion for the rear-end collision model are derived. The model incorporates two vehicles, suspension systems, and a biomechanical representation of the occupant within the front vehicle. Each body and joint are associated with translational and rotational degrees of freedom (3 DOF), as well as with corresponding mass, damping, and stiffness properties. The resulting system of equations, summarized in Table 2, describes the dynamic interaction between the vehicles and the occupant under rear-end impact conditions. In this formulation, each symbol denotes either a kinematic quantity, a physical parameter, or a degree of freedom relevant to the multibody system.

While the model considers a simplified two-vehicle collision scenario, this approach can be extended to more complex events, such as multi-vehicle (mass) crashes, by expanding the dynamic boundary conditions and incorporating additional contact constraints.

This system models the interaction between two vehicles during a rear-end collision and includes a biomechanical model of the human occupant in the front vehicle [31,32,33].

Head:

- Translational motion:
  (2) $$m_{h}{\ddot{x}}_{h}=-k_{hr}\left(x_{h}-x_{hr}\right)-d_{hr}\left({\dot{x}}_{h}-{\dot{x}}_{hr}\right)$$

- Rotational motion:
  (3) $$I_{h}{\ddot{\theta }}_{h}=-k_{\theta ,neck}\left(\theta_{h}-\theta_{N}\right)-d_{\theta ,neck}\left({\dot{\theta }}_{h}-{\dot{\theta }}_{N}\right)$$

Neck:

- Rotational motion (neck stiffness and damping):
  (4) $$I_{N}{\ddot{\theta }}_{N}=k_{H}\left(\theta_{h}-\theta_{N}\right)+d_{H}\left({\dot{\theta }}_{h}-{\dot{\theta }}_{N}\right){-k}_{N}\left(\theta_{h}-\theta_{UB}\right)-d_{N}\left({\dot{\theta }}_{h}-{\dot{\theta }}_{UB}\right)$$

Torso (upper body):

- Translational motion (forces from seatbelt and head–headrest coupling):
  (5) $$m_{UB}{\ddot{x}}_{UB}=k_{sb}\left(x_{2}-x_{UB}\right)+d_{sb}\left({\dot{x}}_{2}-{\dot{x}}_{UB}\right){+k}_{hr}\left(x_{h}-x_{hr}\right)+d_{hr}\left({\dot{x}}_{h}-{\dot{x}}_{hr}\right)$$

- Rotational motion:
  (6) $$I_{UB}{\ddot{\theta }}_{UB}=k_{N}\left(\theta_{N}-\theta_{UB}\right)+d_{N}\left({\dot{\theta }}_{N}-{\dot{\theta }}_{UB}\right){-k}_{UB}\theta_{UB}-d_{UB}{\dot{\theta }}_{UB}$$

Torso (lower body):

- Rotational motion:
  (7) $$I_{LB}{\ddot{\theta }}_{LB}={-k}_{LB}\theta_{LB}-d_{LB}{\dot{\theta }}_{LB}$$

Vehicle 1 (with occupant):

- Translational motion:
  (8) $$M_{1}{\ddot{x}}_{1}={-k}_{s1}x_{s1}-c_{s1}{\dot{x}}_{s1}-k_{sb}\left(x_{1}{-x}_{UB}\right)-d_{sb}\left({\dot{x}}_{1}{-\dot{x}}_{UB}\right){-k}_{c}\left(x_{1}{-x}_{2}\right)-c_{c}\left({\dot{x}}_{1}{-\dot{x}}_{2}\right)$$

Vehicle 2:

- Translational motion:
  (9) $$M_{2}{\ddot{x}}_{2}={-k}_{s2}x_{s2}-c_{s2}{\dot{x}}_{s2}+k_{c}\left(x_{1}{-x}_{2}\right)+c_{c}\left({\dot{x}}_{1}{-\dot{x}}_{2}\right)$$

### 3.4. Active Headrest Implementation

An active headrest system is integrated into the occupant model to lessen the whiplash effect during rear-end collisions. The headrest is actively moved by a linear actuator controlled by a microcontroller that receives data from an accelerometer, in response to detected deceleration. The translational equation of motion for the occupant’s head is increased by the time-dependent force Fact(t) that the actuator applies to the head. As a result, the model can replicate both active and passive whiplash defences (minimizing the head–neck movement during collision).

**Figure 5:**
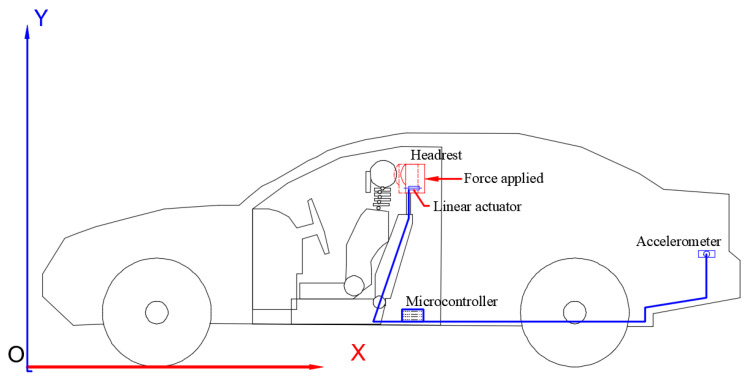
Active headrest schematic.

The vehicle’s rear-end collision-related abrupt deceleration is detected by an accelerometer installed near the bumper or chassis. This signal is processed by the microcontroller, which then turns on the linear actuator that is mounted behind the headrest. By applying a force Fact(t) forward to the headrest, the actuator lessens whiplash by decreasing the relative motion between the head and torso (see Figure 5).

In response to a crash event, the headrest is activated by a linear actuator that is modelled as a prismatic joint with stiffness and damping. It is possible to derive a 1D translational equation of motion (EOM) for the headrest mass along the horizontal axis (X-direction). Using Newton’s Second Law,

(10) $$m_{h}\left({\ddot{x}}_{h}+a_{c}\left(t\right)\right)=-k_{hr}x_{h}-d_{hr}{\dot{x}}_{h}$$

where *m_h_*—is the headrest mass, *k_hr_*—headrest linear stiffness, *d_hr_*—headrest linear damper, and *x_h_*—headrest displacement.

The force applied by the actuator is then

(11) $$F_{act}=-k_{hr}x_{h}-d_{hr}{\dot{x}}_{h}$$

The headrest interaction force that already exists can be increased by the actuator force as follows:

(12) $$m_{h}{\ddot{x}}_{h}=-k_{hr}\left(x_{h}-x_{hr}\right)-d_{hr}\left({\dot{x}}_{h}-{\dot{x}}_{hr}\right)+F_{act}(t)$$

**Figure 6:**
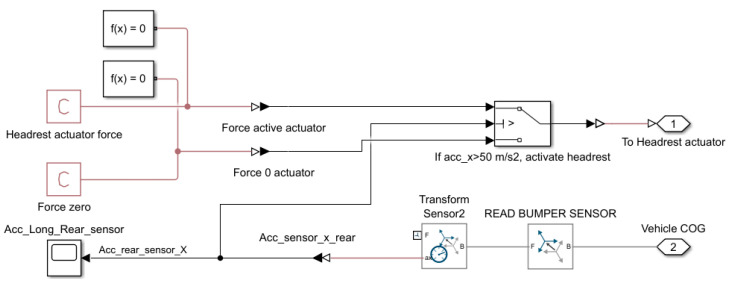
Implementation of active headrest in the Simscape model.

A threshold-based logic that tracks the longitudinal acceleration of the vehicle is used to control the active headrest. When the measured acceleration surpasses a certain threshold (e.g., 50 m/s^2^), a linear actuator applies force to the headrest. This logic is implemented using a Simulink block diagram, as illustrated in Figure 6.

The active headrest system is modelled using a closed-loop Simulink control block. A longitudinal acceleration sensor is placed near the rear of the vehicle (referred to as Acc_Long_Rear_sensor) to detect the sudden deceleration caused by rear-end collisions. The sensor data is transformed into the vehicle’s centre of gravity reference frame using a transform block (Transform Sensor2), and the longitudinal component ax,rear is extracted.

A conditional logic block compares the rear acceleration signal to a threshold value (in this case, 50 m/s^2^). When this threshold is exceeded, a predefined actuator force Fact is applied to the headrest, pushing it forward toward the occupant’s head. If the threshold is not exceeded, the actuator applies zero force.

The headrest force applied to the head can be expressed as follows:

(13) $$F_{act}\left(t\right)=\left\{\begin{array}{r} F_{max},\\ 0, \end{array}\right.\begin{array}{c} if\;a_{x,\;rear}\left(t\right)>a_{thresh}\\ if\;a_{x,\;rear}\left(t\right)<a_{thresh} \end{array}$$

where $a_{x,rear}\left(t\right)$—longitudinal acceleration measured at the rear of the vehicle, $a_{thresh}=50\;m/s^{2}$—activation threshold and *F_max_*—constant actuator force.

### 3.5. Simscape Model

**Figure 7:**
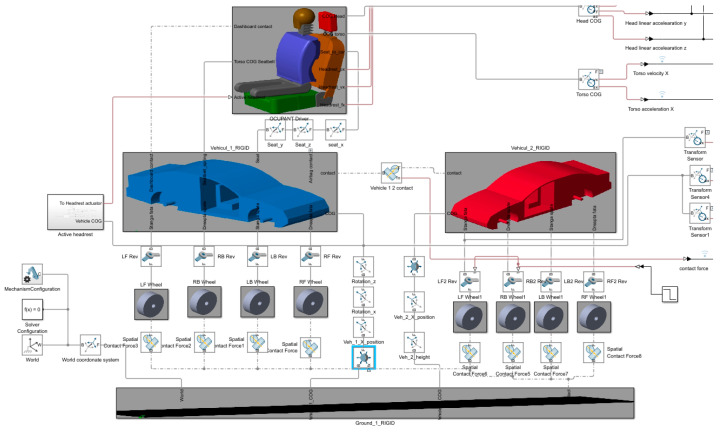
Simscape model for rear-end collisions.

The functional simulation model was developed using Simscape Multibody in MATLAB. CAD-derived parts were imported and interconnected using mechanical joints to accurately represent the dynamic behaviour of the system during a rear-end collision.The assembled Simscape model, illustrated in Figure 7, is structured into three principal components: Vehicle 1 (depicted in blue), Vehicle 2 (depicted in red), and the occupant positioned within Vehicle 1.

Each principal component is organized into a hierarchical subsystem mask containing multiple layers of individual elements, such as wheels, suspension components, body structures, and occupant segments. All components are connected through defined reference points and a combination of prismatic, revolute, and translational joints, ensuring accurate kinematic and dynamic interaction between the bodies.

This modular architecture allows for clear model organization, facilitates parameter updates, and provides the flexibility to adapt the system for future extensions or additional active safety systems.

Figure 7 illustrates the complete Simscape Multibody model developed for the simulation of rear-end collisions. The base body is connected to the real-world frame and serves as the global reference for all bodies in the system. Both vehicles are modelled with joints connecting their chassis to their respective wheels, allowing for independent suspension behaviour. The interaction between the vehicles and the ground is modelled by introducing spatial contact forces at each wheel, simulating tyre–ground contact and enabling the transmission of vertical and longitudinal forces during impact and rebound.

An additional contact force is defined between the front of Vehicle 1 and the rear of Vehicle 2, representing the stiffness and damping characteristics of the front-end structures during collision.

To enable realistic vehicle motion,

- Vehicle 1 is connected to the ground through a 6-DOF joint, allowing for full three-dimensional movement with controlled constraints;
- Vehicle 2 is connected to the ground via a prismatic joint, permitting only longitudinal translation, with the initial velocity of Vehicle 2 adjustable to simulate different rear-end impact scenarios.

Within Vehicle 1, the occupant seat and chassis are connected by a prismatic joint combined with a spring–damper system to model the dynamic tension and restraint effect of the seatbelt during collision.

The neck segment of the occupant is connected to the upper body via a revolute joint and a spatial contact force, allowing for a realistic head–torso relative motion and enabling the simulation of neck flexion under impact conditions.

This model architecture ensures the accurate representation of vehicle and occupant dynamics, structural compliance, and contact interactions necessary for simulating rear-end crash scenarios involving active safety systems. The contact force between the components was calculated using Equation (2) [32,34,35].

(14) $$f_{n}=s(d)\cdot (k\cdot d+b\cdot \dot{d})$$

where

- *f_n_* is the normal force applied in equal-and-opposite fashion to each contacting geometry.
- *d* is the penetration depth between two contacting geometries.
- *d’* is the first-time derivative of the penetration depth.
- *k* is the normal-force stiffness specified in the block.
- *b* is the normal-force damping specified in the block.
- *s*(*d*) is the smoothing function.

A low-pass filter with a cut-off frequency of X Hz was used as the smoothing function to eliminate high-frequency noise from the sensor signal.

**Figure 8:**
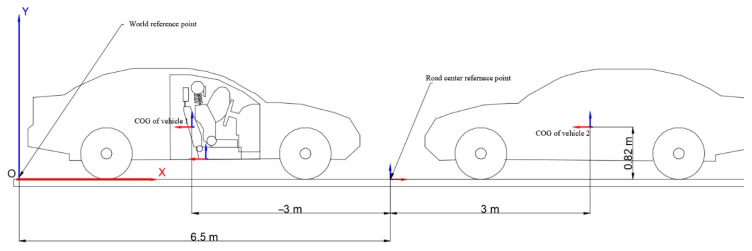
Vehicle position with respect to the real-world reference frame.

The position of the vehicles is shown in Figure 8. This representation shows the real-world frame of reference and the position of the two vehicles in relation to this frame of reference. This is crucial when analyzing the displacement parameters of individual components of the model. Note that the road reference point is positioned with respect to the real-world reference point and the vehicles are positioned with respect to the road reference point.

### 3.6. Model Parameters

In this section, the mathematical parameters for the model are presented and grouped into categories. These parameters define the functionality of the model and ensure that the model has a dynamic and kinematic response identical to real-world situations and multiple types of crash configurations. The first set of parameters are the mass parameters for each CAD component. These were used to calculate the inertia and contact forces during a crash (see Table 3).

1. Mass parameters

The values chosen for the model are the average for each component. Vehicle 1 has a lower mass due to the interior bodies such as occupant, dashboard, and seat. The occupant weighs a total of 71 kg, which is the average for a man in the 50th percentile range. The mass values were average and identical to those used in the experimental tests to validate the model. The second set of parameters are the dynamic ones, such as velocities and gravitational acceleration (see Table 4).

2. Dynamic parameters

For validation purposes, Vehicle 1 had a speed of 0 km/h and Vehicle 2 had a speed between 33 and 35 km/h (33 km/h for the rear impact and 35 km/h for the frontal impact). Both vehicles have a positive caster angle of 4 degrees. Although Vehicle 1 is modelled as being stationary in this scenario, the resulting relative velocity simulates the real-world condition where the leading vehicle decelerates and the following driver fails to brake in time —a common cause of rear-end collisions in urban traffic. The third set of parameters are the spring values of the joints. There are 3 revolute joints on the occupant and a prismatic joint for the vehicle suspension. The occupant seatbelt is a linear spring. The values are shown in Table 5.

3. Spring and damper parameters

The seatbelt connection is a linear spring that connects two reference points: one on the vehicle and one on the occupant. The values were determined in previous studies and according to standards [36,37,38].

The vehicle suspension stiffness and damping values correspond to previous research and standards related to vehicle dynamics [38,39,40].

Previous research shows that average neck stiffness is between 0.5 Nm/rad and 2 Nm/rad (0.03 Nm/deg), determined using biomechanical analyses. However, in the presented model, using such low values are not enough to keep the head in the upright position. By adjusting the stiffness and running the simulation several times, it was observed that the optimal value of neck stiffness is around 86 Nm/rad (approx. 1.5 Nm/degree). This value ensures the correct kinematic response compared to the crash test dummy used in the experiment [41,42,43,44,45].

The neck-damping values were also determined from biomechanical analyses, and are between 0.1 and 0.5 Nm/rad (0.017 Nm/deg). Here, too, running the simulation several times resulted in an optimal value of 0.02 for the model presented [46,47,48,49]. The fourth set of parameters are the connection types and degrees of freedom between them. These are presented in Table 6.

4. Connections between the model bodies

The last set of parameters are the contact stiffness and damping between the bodies that have contact with each other. These are presented in Table 7.

5. Contact parameters

The stiffness of the vehicle and the damping were determined experimentally and analytically. To better understand this, the absorbed kinetic energy equation can be used for the vehicle stiffness calculation [50]:

(15) $$E_{k}=\frac{1}{2}mv^{2}$$

where m is the vehicle mass (kg) and v is the collision velocity (m/s).

Stiffness is determined by the total deformation of the front surface of the vehicle; therefore, we can denote it as *D_x_*. During collision, the kinetic energy is converted into elastic potential energy, which can be calculated using Equation (4).

(16) $$E_{p}=\frac{1}{2}k{\left(∆x\right)}^{2}$$

If the initial kinetic energy becomes potential energy, we use the following equation:

(17) $$\frac{1}{2}mv^{2}=\frac{1}{2}k{\left(∆x\right)}^{2}$$

From Equation (5), we can determine the rigidity value *k* (N/m) in Equation (6):

(18) $$k=\frac{mv^{2}}{{\left(∆x\right)}^{2}}$$

The value of the stiffness can differ from one case to another because of the frontal deformation value of each vehicle. The average value of k can be calculated by determining an average deformation value from experimental controlled crash tests or real vehicle crashes for a given velocity and one type of vehicle class. For the given model, the vehicle is a sedan type and the impact velocity is around 35 km/h (11.1 m/s). Documentation regarding the deformation values for frontal collision, known as the EES catalogue, can give us the possibility to determine the average deformation for a sedan-class vehicle at the equivalent energy speed (EES) of 15 km/h, which is about 35 km/h impact velocity, after the energy absorption is considered. The deformation values are presented in Table 8 [51,52].

The damping value for the model was determined using the natural frequency and critical damping equations [50].

(19) $$\omega_{n}=\sqrt{\frac{k}{m}}$$

where *w_n_* is the natural frequency (rad/s), k is the stiffness, and m is the mass. To calculate the critical damping value *c_c_*, the following equation can be used:

(20) $$c_{c}=2\sqrt{km}$$

Depending on the vehicle type and class, the contact between vehicles may be modified to better adapt to the frontal stiffness of each vehicle.

### 3.7. Model Dimensions

The vehicle is a standard sedan class. The measurements are average values for this class [50]. Depending on one’s needs, these models can be modified and adapted for different crash scenarios. The occupant and seat have the dimensions of an average 50th percentile male body [53,54].

To simplify the representation of the crash phenomenon, the occupant was shown in a simplified manner without limbs. However, this simplification has no influence on the kinematics of the body in a frontal or rear-end crash.

## 4. Model Validation

To validate the virtual model and demonstrate that the output results of the model are correct and reflect the outcome of an accident, experimental tests were conducted in a controlled environment using average passenger vehicles and crash test dummies.

To quantitatively validate the virtual model against experimental crash test data, RMSE, MAPE, and MAE indicators were calculated using the next set of equations [55,56,57,58].

(21) $$RMSE=\sqrt{\frac{1}{n}\sum_{i=1}^{n}{\left(y_{i}^{sim}-y_{i}^{test}\right)}^{2}}$$

(22) $$MAPE=\frac{100\%}{n}\sum_{i=1}^{n}\left|\frac{y_{i}^{sim}-y_{i}^{test}}{y_{i}^{test}}\right|$$

(23) $$MAE=\frac{1}{n}\sum_{i=1}^{n}\left|y_{i}^{sim}-y_{i}^{test}\right|$$

These statistical indicators were used to assess the level of agreement between the simulated outputs and experimental crash test data. According to commonly accepted validation criteria in occupant safety modelling, an average error below 5% is considered an indicator of good model fidelity. In this study, all three metrics—RMSE, MAPE, and MAE—were calculated using the resultant acceleration data of the head. The results confirm that the differences between the model and the experimental data remained within an acceptable threshold, thus validating the accuracy and reliability of the virtual multibody system.

### 4.1. Frontal Collision

**Figure 9:**
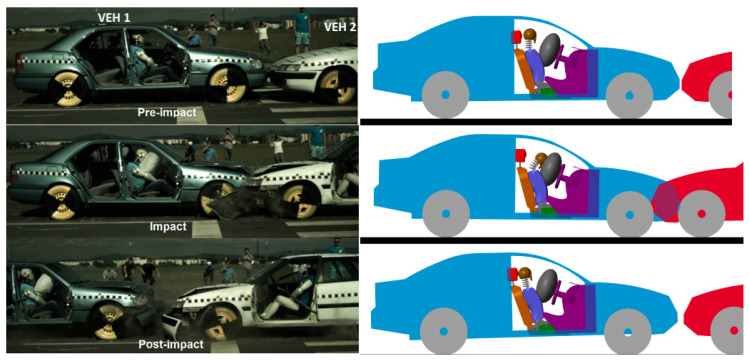
Kinematic comparison of the virtual model and the experimental test for a frontal collision.

The first validation concerned a frontal collision at 35 km/h with an airbag. Vehicle 1 was stationary, and the second vehicle accelerated, and the collision occurred with an overlap of 100%. The kinematics and impact phases are shown in Figure 9.

We can observe that, in the pre-impact phase, in a side-by-side comparison, the occupant and the vehicle perfectly match the position of the occupant of the experiment. In the impact phase, the kinematics of the occupant corresponds to the kinematics of the crash test dummy. The same applies to the post impact phase.

**Figure 10:**
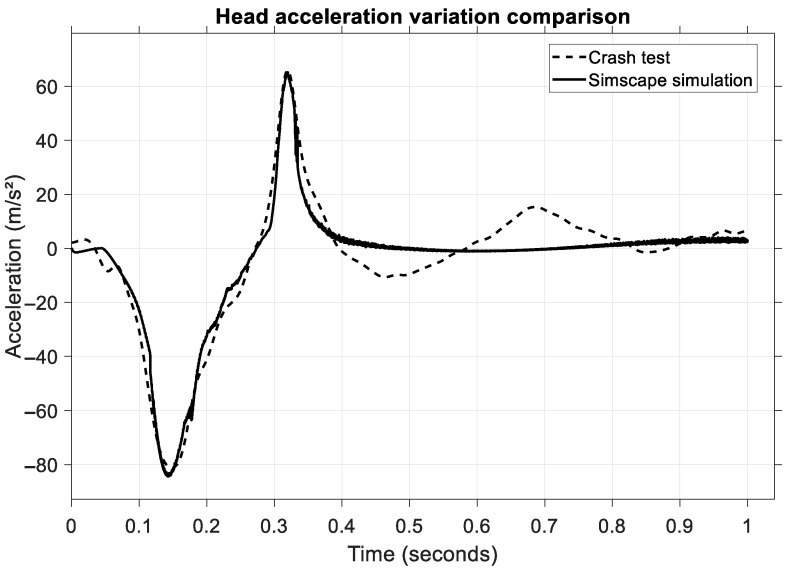
Comparison of head acceleration between the virtual model and the experimental test.

To validate the model numerically, a comparison of vehicle velocities and head acceleration values was carried out. The first result is the head acceleration, shown in Figure 10.

It can be observed that both acceleration values match, almost perfectly overlapping each other, granting a high level of similarity. In this type of collision, the acceleration is on the *X*-axis, the longitudinal axis of the vehicle, and two phases can be distinguished on the diagram: the head and neck flexion phase and the extension phase. The first phase lasts between 0.1 and 0.25 s, while the second phase lasts between 0.25 and 0.45 s. These values (80 m/s^2^ and 65 ms^2^, respectively) are low in terms of occupant injury due to two factors: the belt tensioner and the front airbag.

To quantitatively assess the agreement between the simulation results and the crash test data, three statistical indicators were calculated: Root Mean Square Error (RMSE), Mean Absolute Percentage Error (MAPE), and Mean Absolute Error (MAE). The results for head acceleration during the frontal collision scenario are summarized in Table 9.

The low values obtained, particularly the MAPE of 0.71%, indicate a high level of accuracy between the simulated and experimental data. This confirms the validity of the multibody model in predicting occupant head dynamics under frontal collision conditions.

**Figure 11:**
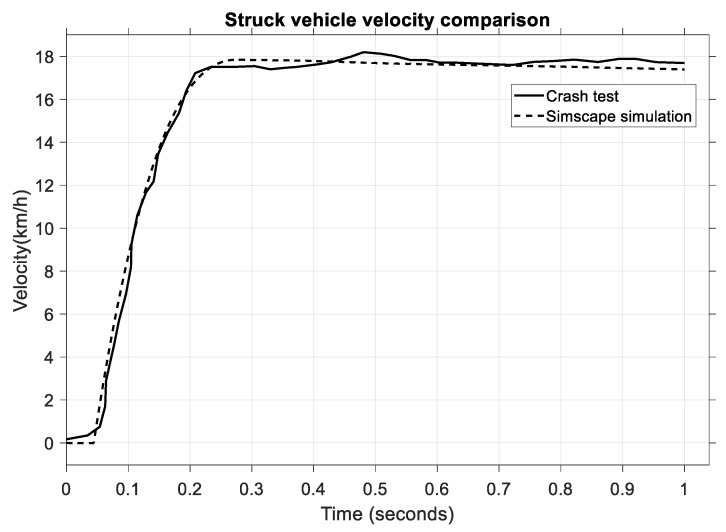
Comparison of vehicle velocities for the struck vehicle.

In the next figure, Figure 11, a comparison is made between the struck vehicle velocity.

Both the crash test and the model have good similarity, which ensures the high accuracy of the mathematical model, although there is a slight difference of 0.3 s–0.9 s in the post-impact velocity. What is crucial here, however, is the impact phase between 0.05 and 0.3 s, in which an increase in velocity from 0 km/h to 18 km/h can be observed. To further strengthen the validity of the virtual model, a statistical comparison of the struck vehicle’s velocity was also performed using RMSE, MAPE, and MAE indicators.

The results in Table 10 indicate a very high level of agreement between the simulated and experimental vehicle velocities, with all error metrics well below the 5% threshold, confirming the accuracy of the model’s dynamic response during impact.

**Figure 12:**
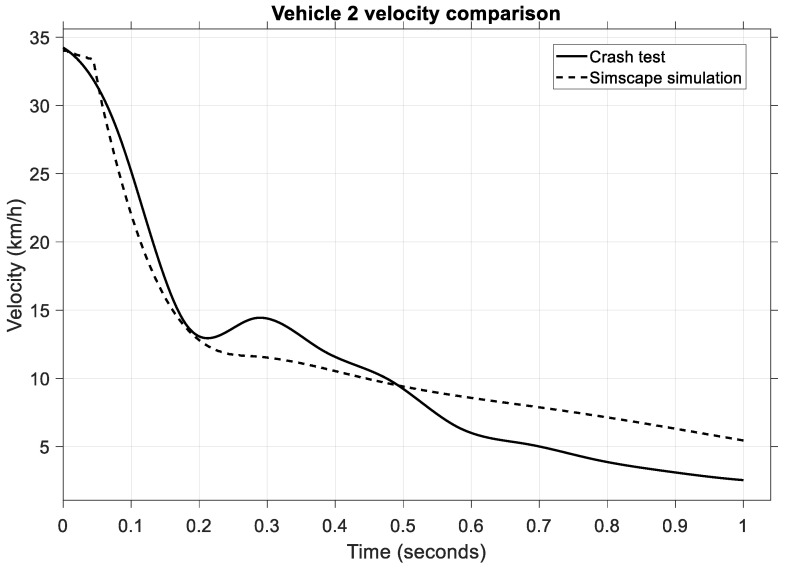
Comparison of vehicle velocities for the striking vehicle.

A similar velocity variation occurs for the second vehicle, the impact vehicle, whose speed is shown in Figure 12.

The velocity for the striking vehicle starts at 35 km/h, and the impact velocity for the crash test and the variation is observed to follow a similar timeframe as the struck vehicle, from 0.05 to 0.25 s. Even though these plots do not overlap perfectly, the variation during the critical phase, such as the impact phase, is almost identical, and this is the important aspect of the validation process for the mathematical model.

### 4.2. Rear-End Collision

**Figure 13:**
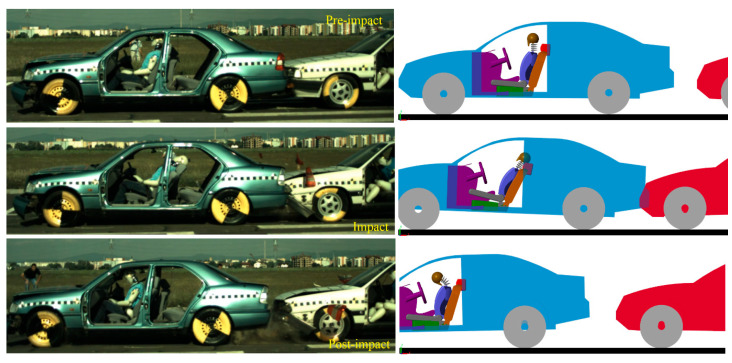
Kinematic comparison of the virtual model and the experimental test for the rear-end collisions.

The model can be easily configured for rear-end collisions by simply rotating the struck vehicle 180 degrees on the *Z*-axis. In such a scenario, the occupant’s kinematics follow a similar pattern to frontal collisions, only this time, the headfirst contacts the headrest and then swings forward for the flexion phase. The validation of this crash was also achieved by comparing the kinematic and dynamic values of a real crash test, shown in Figure 13. The crash test was carried out at 33 km/h with the same vehicles and the same dummy as in the frontal impact with identical parameters. This comparison validates the mathematical model for rear-end collisions by comparing the kinematic and dynamic values.

**Figure 14:**
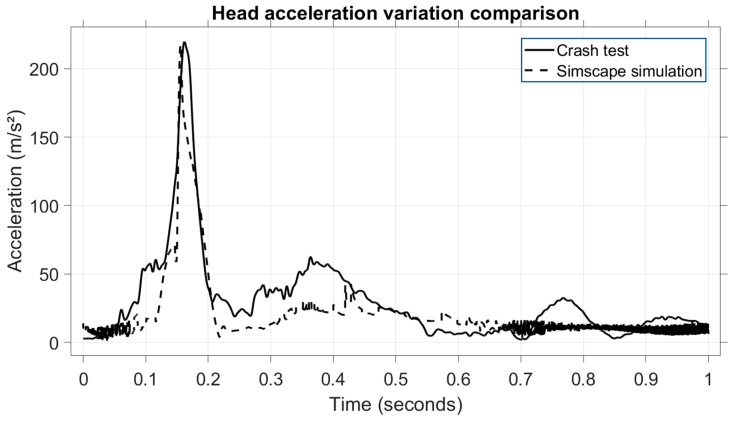
Comparison of head acceleration between the virtual model and the experimental test for the second scenario.

Here, too, the kinematic comparison was carried out using the crash test and the model, which are compared side-by-side to better observe the occupant’s kinematics. It can be clearly seen, in both the model and the crash test, that the occupant’s head hits the headrest during the impact phase, and the head swings forward in the post-impact phase. For numerical validation, in Figure 14, the head acceleration values are presented. For this test, the resultant acceleration is used.

In this scenario, the impact phase occurs between 0.1 and 0.2 s when the head hits the headrest, and a maximum head deceleration value of 200 m/s is reached. Both the crash test and the model have an almost identical pattern during the impact phase, thus ensuring a high level of accuracy for the model created. To support this observation quantitatively, statistical indicators were computed. Table 11 presents the RMSE, MAPE, and MAE values for the rear-end collision head acceleration comparison.

**Figure 15:**
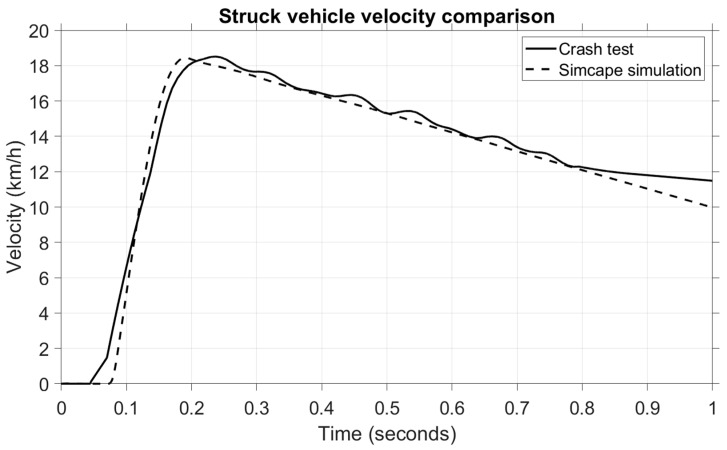
Comparison of vehicle velocities for the struck vehicle—second scenario.

To further analyze this scenario, the speed of the struck vehicle is shown in Figure 15.

We can see that the velocity of the struck vehicle increases from 0 to 18 km/h within 0.1–0.2 s, and the timeframe is almost identical to the head acceleration variation during the impact phase. To support this result quantitatively, Table 12 presents the statistical parameters (RMSE, MAPE, and MAE) used to evaluate the agreement between the experimental crash data and the simulation results. All indicators fall well below the 5% error threshold, confirming the reliability of the model in replicating the kinematic behaviour of the struck vehicle.

**Figure 16:**
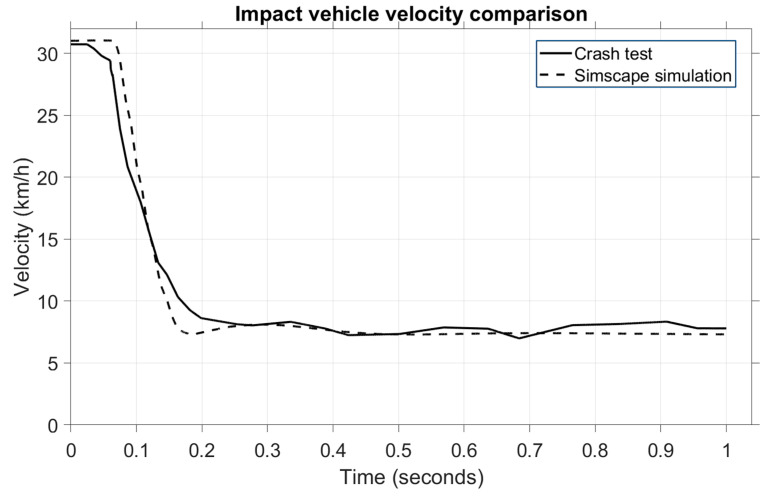
Comparison of vehicle velocities for the impact vehicle—second scenario.

In Figure 16, we can observe the velocity comparison of the impact vehicle.

In this case, a change in velocity is observed in the same period of 0.1–0.2 s when the value drops from 34 km/h to 5 km/h during the impact phase. Note that the model and the crash test only show an almost identical pattern in the mentioned time. In the post-impact phase, the crash test vehicle continued to roll at 15 km/h, while in the model, the velocity dropped to 5 km/h in the next 0.8 s. This is because, in the model, the brakes were applied, while in the crash test, the car rolled another second before the brakes were activated.

## 5. Active Headrest Simulation

The effectiveness of an active headrest in a rear-end collision was assessed using the validated dynamic model of the occupant–vehicle system. Three distinct collision speeds were modelled:

- 20 km/h (low severity);
- 31 km/h (validation speed, based on experimental data);
- 40 km/h (high severity).

**Figure 17:**
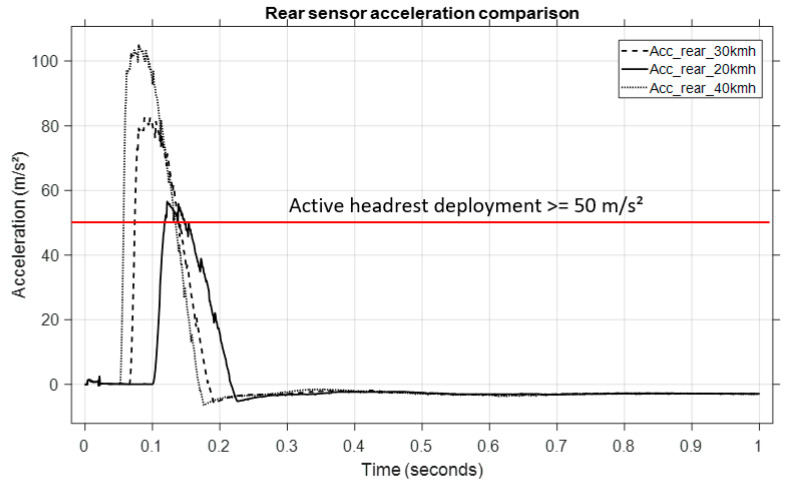
Struck vehicle acceleration comparison at different velocities.

A virtual sensor placed close to the rear bumper, which mimicked the actual vehicle sensor, was used to measure the rear acceleration. Figure 17 displays the longitudinal acceleration profiles that were captured at these speeds, with reference to the initial plot. One can observe the following:

The maximum rear acceleration increases with collision severity.

Every velocity causes the acceleration to approach or surpass values near 50 m/s^2^, which was chosen as the active headrest system’s activation threshold.

The active headrest system was triggered when the measured rear acceleration exceeded 50 m/s^2^, according to the logic described previously. Upon activation, a predefined force was applied to the headrest to push it forward and reduce the head–torso relative displacement (whiplash effect).

Without activation, the head moved backward freely relative to the torso (normal headrest scenario).

With activation, the headrest moved forward synchronously, limiting relative motion (active headrest scenario).

**Figure 18:**
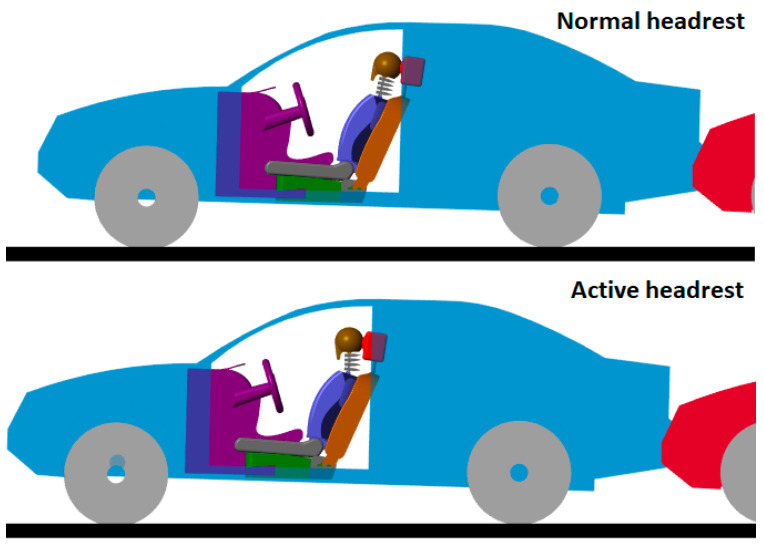
Kinematic comparison of the occupant with and without the active headrest.

Figure 18 (second image) shows a comparison of occupant kinematics during the collision:

Normal headrest (top figure):

- Large backward motion of the head relative to the torso.
- Neck hyperextension occurred.

Active headrest (bottom figure):

- Head motion relative to the torso was reduced.
- Neck bending was significantly limited, minimizing injury risk.

The active headrest effectively reduced the whiplash effect by proactively supporting the head during the critical early phase of the collision (approximately 0.1–0.2 s after impact).

**Figure 19:**
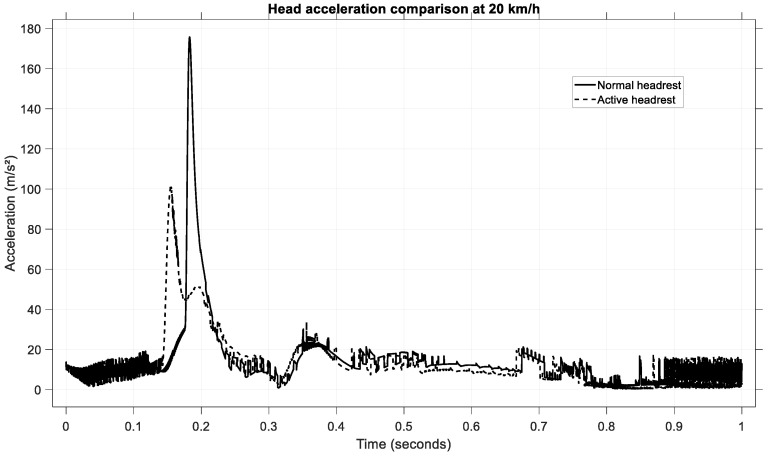
Head acceleration comparison at 20 km/h.

To further assess the effectiveness of the active headrest, the head linear acceleration was analyzed for a low-speed rear-end collision at 20 km/h. Figure 19 presents the time histories of the head acceleration for both configurations: passive (normal) headrest and active headrest.

In the passive headrest case, the occupant’s head experienced a sharp acceleration peak of approximately 175 m/s^2^, occurring around 0.20 s after impact initiation.

By contrast, when the active headrest was deployed, the peak head acceleration was significantly reduced to approximately 110 m/s^2^. Additionally, the active headrest led to a broader and smoother acceleration pulse, indicative of a more gradual deceleration of the head.

This reduction of approximately 37% in peak acceleration highlights the effectiveness of the active system in mitigating injurious head motions. Moreover, the earlier engagement of the headrest (approximately at 0.17 s) demonstrates the benefit of anticipative movement control, ensuring head support during the critical whiplash phase.

**Figure 20:**
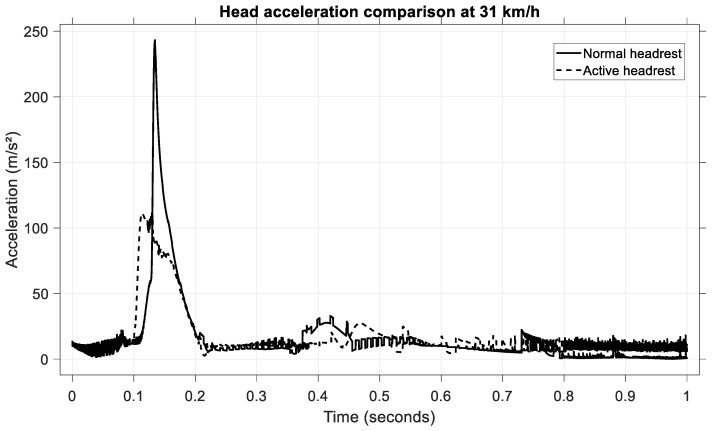
Head acceleration comparison at 31 km/h.

To validate the model under a medium-severity rear-end collision, simulations were performed at an impact speed of 31 km/h. The resulting head acceleration profiles for the passive and active headrest configurations are shown in Figure 20.

In the case of the passive headrest, the occupant’s head experienced a pronounced acceleration peak, reaching approximately 240 m/s^2^ and occurring at around 0.14 second’s post-impact.

Conversely, when the active headrest was engaged, the maximum head acceleration was limited to approximately 105 m/s^2^. The active configuration also resulted in a more gradual acceleration profile, characterized by a reduced rate of change (jerk) and a broader temporal distribution of the peak.

This corresponds to a reduction of approximately 56% in the peak head acceleration when using the active headrest. Such a significant reduction confirms the capacity of the active headrest to mitigate the head’s backward motion, thereby diminishing the loads transmitted through the cervical spine.

**Figure 21:**
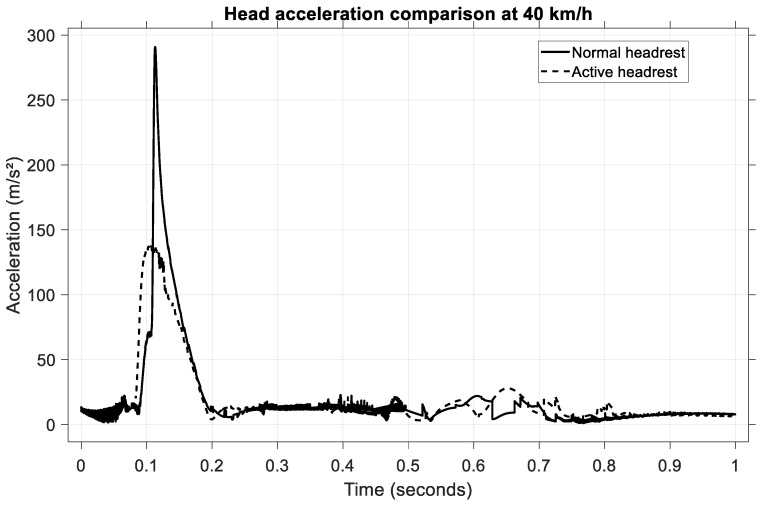
Head acceleration comparison at 40 km/h.

To assess the performance of the active headrest under high-severity impact conditions, a rear-end collision at 40 km/h was simulated. The head acceleration time histories for both passive and active headrest configurations are illustrated in Figure 21.

In the case of the normal (passive) headrest, the occupant’s head exhibited a sharp acceleration peak, reaching approximately 290 m/s^2^ and occurring at approximately 0.12 s following impact. When the active headrest was employed, the peak head acceleration was significantly attenuated, reaching a value of approximately 135 m/s^2^. Additionally, the shape of the acceleration curve under active control is smoother, indicating reduced jerk levels and a more progressive head deceleration.

The active system therefore achieved a reduction of approximately 53% in the peak head acceleration compared to the passive configuration. This substantial improvement in head kinematic control, particularly under severe impact conditions, confirms the effectiveness of the active headrest in mitigating potential whiplash injuries by providing earlier and more controlled head support.

The numerical simulations conducted at 20 km/h, 31 km/h, and 40 km/h rear-end collision scenarios demonstrate the significant benefits of the active headrest system in mitigating head kinematics and reducing injury risk. Across all impact speeds, the active headrest effectively reduced the peak head acceleration by approximately 37%–56% depending on collision severity. Additionally, the active system enabled earlier and smoother deceleration of the head, limiting relative motion between the head and torso and thereby minimizing neck extension.

These results confirm that the implementation of an active headrest mechanism offers a substantial improvement in occupant protection compared to a conventional passive system, particularly during moderate- and high-severity rear-end impacts.

These findings support the use of active headrest technologies as a viable countermeasure for reducing whiplash-associated injuries in automotive collisions.

## 6. Discussion and Conclusions

This study presents the development of a comprehensive mathematical model using a multibody system (MBS) approach to analyze the dynamics of motor vehicles during frontal and rear-end collisions. By integrating rigid body dynamics, occupant biomechanics, vehicle suspension behaviour, and advanced modelling of restraint systems, the model provides a robust and flexible framework for crash analysis, including the design and validation of an active headrest system with integrated sensing.

A key innovation introduced in this work is the inclusion of an active headrest system, which is controlled via an integrated longitudinal acceleration sensor and a dedicated logic circuit. Upon detecting a rear-end impact exceeding a defined acceleration threshold, the system actively displaces the headrest to limit head–torso relative motion. Simulation results across various collision speeds (20, 31, and 40 km/h) confirmed the effectiveness of the active headrest in significantly reducing peak head accelerations and mitigating potential whiplash injuries.

The model was validated against experimental crash test data, demonstrating excellent agreement in both vehicle kinematics and occupant dynamics. The ability of the model to replicate complex interactions between vehicle structures, restraint systems, and occupant body segments confirms its reliability and applicability for real-world automotive safety research and vehicle design optimization.

The validation scenarios in this study focus on rear-end collisions at low to medium speeds (≤40 km/h), which represent the majority of real-world rear-end impacts. At these speeds, occupant kinematics and acceleration profiles are predominantly influenced by seat restraint systems and headrest geometry, rather than extensive structural deformation. Also, the current validation includes collisions up to 40 km/h, focusing on urban scenarios, where whiplash injuries are most prevalent. Future work will involve extending the model to higher-speed crashes (e.g., 60 km/h) and coupling it with vehicle deformation models.

The presented model focuses on occupant dynamics without explicit structural deformation, a simplification which has been noted in the previous literature. For example, Przemysław P et al. [59] studied vehicle structural deformation and its effect on energy absorption and occupant deceleration using finite element models. While our multibody model cannot capture local deformation effects, it provides accurate timing and magnitude of head acceleration and body motion, which are critical for early whiplash prediction. The timing of peak head acceleration (~200 m/s^2^ at ~0.18 s) aligns well with the values reported in validated rear-end impact simulations [60].

In addition to its predictive accuracy, the developed model offers substantial practical advantages. A MATLAB-based control script enables users to easily configure simulation parameters, run the Simulink model, and automatically export results to a spreadsheet format. This streamlined process enhances the model’s accessibility and usability, allowing for rapid iteration and scenario testing without the need for extensive manual intervention.

The current model is based on a 50th percentile male occupant. Biomechanical variations associated with different occupant groups, such as females or children, were not considered. However, the parametric flexibility of the Simscape Multibody platform allows future inclusion of demographic variability through modifications of joint properties, mass distribution, and anthropometric dimensions.

Nevertheless, the model does present some limitations. It does not explicitly simulate large material deformations in components such as airbags, seats, or the vehicle’s crumple zones. These phenomena, characterized by complex nonlinear behaviour, require finite element modelling (FEM) approaches, which were beyond the scope of the present study. However, it should be noted that the force and acceleration interactions between bodies are correctly computed based on equivalent stiffness and damping parameters, ensuring that dynamic responses remain physically meaningful, even without visual deformation.

Future research directions may involve extending the model to incorporate different impact configurations (e.g., oblique or lateral impacts), a wider variety of vehicle types, and more detailed interior component modelling. Furthermore, integrating additional active safety devices, such as pre-crash seatbelt systems or adaptive airbags, could enhance the model’s capability to evaluate next-generation automotive safety technologies.

Overall, the development and validation of this multibody system model highlights the importance of multidisciplinary methodologies in advancing vehicle safety research. By combining MBS dynamics, integrated sensor-based control strategies, and experimental validation, this work establishes a solid foundation for future innovations in occupant protection and crash-mitigation systems.

## Figures and Tables

**Table 1:** Figure notations.

Notation	Description
1	Rigidity between the head and upper seat (headrest)
2	Rigidity between the head and airbag
3	Rigidity between the wheels and the car (suspension)
4	Rigidity between the upper torso and the upper seat
5	Rigidity between the car and the upper body (seatbelt)
6	Rigidity between the lower body and the lower seat (lower seatbelt)
7	Torsion springs between the upper body and the lower body
8	Torsion spring in the neck
9	Rigidity between the wheels and the ground

**Table 2:** Model parameters.

Notation	Description
*x*1	Position of Vehicle 1 (rear vehicle)
*x*2	Position of Vehicle 2 (front vehicle)
*xs*1	Suspension deflection of Vehicle 1
*xs*2	Suspension deflection of Vehicle 2
*x_UB_*	Translational position of the upper body (torso)
*θ_UB_*	Rotation angle of the upper body (torso)
*x_LB_*	Translational position of the lower body
*θ_LB_*	Rotation angle of the lower body
*k_LB_*	Rotational stiffness of the lower body
*d_LB_*	Rotational damping of the lower body
*θ_H_*	Rotation angle of the head
*θ_N_*	Rotational angle of the neck
*x__H_*	Position of the head
*x__hr_*	Position of the headrest (in Vehicle 2)
*k__H_*	Head–neck stiffness
*d__H_*	Head–neck damping
*k__N_*	Neck-torso stiffness
*d__N_*	Head–torso damping
*m__h_*	Mass of the head
*m_UB_*	Mass of the upper body (torso)
*M*_1_	Mass of Vehicle 1
*M*_2_	Mass of Vehicle 2
*F_act_*	Active headrest actuator force
*I_h_*	Moment of inertia of the head
*I_N_*	Moment of inertia of the neck
*I_ub_*	Moment of inertia of the upper body (torso)
*k_hr_*	Headrest stiffness
*d_hr_*	Headrest damping coefficient
*k_sb_*	Seatbelt stiffness
*d_sb_*	Seatbelt damping coefficient
*k_s_*_1_	Suspension stiffness of Vehicle 1
*c_s_*_1_	Suspension damping coefficient of Vehicle 1
*k_s_*_2_	Suspension stiffness of Vehicle 2
*c_s_*_2_	Suspension damping coefficient of Vehicle 2
*k_c_*	Stiffness of the inter-vehicle contact
*c_c_*	Damping of the inter-vehicle contact
*k_θ_neck_*	Neck joint stiffness
*d_θ_neck_*	Neck joint damping
*k_θ_ub_*	Seatback stiffness (torso rotation)
*d_θ_ub_*	Seatback damping (torso rotation)
*L*	Effective distance from upper body to head centre
*l_hr_*	Offset from vehicle 2 reference to headrest

**Table 3:** Model mass parameters.

Body	Parameter	Mass Value	Unit
Vehicle 1	*M*_1_	1236	Kg
Vehicle 2	*M*_2_	1450	Kg
Wheel × 4	*M_w_*	20	Kg
Dashboard	*M_dhb_*	50	Kg
Airbag	*M_arb_*	0.5	Kg
Lower seat	*M_ls_*	46	Kg
Upper seat	*M_us_*	35	Kg
Headrest	*M_hr_*	3	Kg
Lower body	*M_LB_*	25	Kg
Upper body	*M_UB_*	40	Kg
Neck × 4	*M_n_*	0.2	Kg
Head	*M_h_*	5	Kg

**Table 4:** Model dynamic parameters of the model.

Parameter	Parameter	Value	Unit
Vehicle 1 velocity	${\dot{x}}_{1}$	0	Km/h
Vehicle 2 velocity	${\dot{x}}_{2}$	33–35	Km/h
Gravitational acceleration	g	9.81	m/s^2^
Front suspension caster angle	$\alpha_{c}$	4	degrees

**Table 5:** Spring parameters.

Bodies	Type of Joint	Param.	Stiffness	Unit	Damping	Unit
Head–neck	Revolute	k	1.5	Nm/deg	0.02	Nm/(deg/s)
Upper body–Lower body	Revolute	*k_U_*	0.5	Nm/deg	0.05	Nm/(deg/s)
Upper seat–Lower seat	Revolute	*k_S_*	200	Nm/deg	10	Nm/(deg/s)
Vehicle suspension	Prismatic	*k_sus_*	30,000	N/m	3000	N/(m/s)
Seatbelt	Linear spring	*k_st_*	50,000	N/m	1000	N/(m/s)

**Table 6:** Connection types of the model.

Bodies	Connection Type	Degrees of Freedom
World–Road surface	Fixed joint	0
Vehicles–Road surface	Six-degrees-of-freedom joint	6
Vehicle–Wheels	Prismatic joint	1
Wheels–Vehicle	Revolute joint	1
Dashboard–Vehicle 1	Fixed joint	0
Lower seat–Vehicle 1	Fixed joint	0
Lower seat–Upper seat	Revolute joint	1
Lower seat–Lower body	Six-degrees-of-freedom joint	6
Lower body–Upper body	Revolute joint	1
Headrest–Upper seat	Fixed joint	0
Upper body–Neck	Revolute joint	1
Head–Neck	Revolute joint	1
Model degrees of freedom		4

**Table 7:** Model contact parameters.

Bodies in Contact	Stiffness	Unit	Damping	Unit
Wheels–Road surface	100,000	N/m	100,000	N/(m/s)
Vehicle 1–Vehicle 2	500,000	N/m	80,000	N/(m/s)
Head–Airbag	8000	N/m	10	N/(m/s)
Head–Headrest	8000	N/m	100	N/(m/s)
Head–Upper seat	10,000	N/m	1000	N/(m/s)
Head–Upper body	10,000	N/m	1000	N/(m/s)
Lower body–Lower seat	10,000	N/m	1000	N/(m/s)
Head–Dashboard	100,000	N/m	1000	N/(m/s)
Upper body–Dashboard	100,000	N/m	1000	N/(m/s)

**Table 8:** Deformation values for frontal collisions from the EES catalogue [51,52].

Impact Velocity [km/h]	EES [km/h]	Deformation [m]	Type of Collision
35	15	0.18	Frontal
35	15	0.19	Frontal
35	15	0.20	Frontal
35	15	0.21	Frontal
35	15	0.21	Frontal
35	15	0.22	Frontal
35	15	0.22	Frontal
35	15	0.23	Frontal
35	15	0.25	Frontal
35	15	0.26	Frontal
Average		**0.217**	

**Table 9:** Statistic parameter for the head acceleration in frontal collision.

Parameter	Value	Unit
RMSE	4.19	m/s^2^
MAPE	0.71	%
MAE	4.19	m/s^2^

**Table 10:** Statistic parameters for the struck vehicle velocity comparison.

Parameter	Value	Unit
RMSE	0.29	m/s
MAPE	0.01	%
MAE	0.29	m/s

**Table 11:** Comparison of the statistic parameters of head acceleration for the rear-end collisions.

Parameter	Value	Unit
RMSE	0.14	m/s^2^
MAPE	0.01	%
MAE	0.14	m/s^2^

**Table 12:** Comparison of the statistic parameters of vehicle velocity for the rear-end collisions.

Parameter	Value	Unit
RMSE	1.39	m/s
MAPE	0.12	%
MAE	1.39	m/s

## Data Availability

Data are contained within the article.

## Abbreviations

The following abbreviations are used in this manuscript:

MBS	Multibody System
CAD	Computer Aided Design
MEMS	Micro-Electro-Mechanical Systems
ODE	Ordinary Differential Equations
EOM	Equation of Motion
RMSE	Root Mean Square Error
MAE	Mean Absolute Error
MAPE	Mean Absolute Percentage Error
MATLAB	Matrix Laboratory
DOF	Degrees of Freedom
EES	Equivalent Energy Speed
1D	One Dimension
